# Health Impact and Cost-Effectiveness of Implementing Gender-Neutral Vaccination With the 9-Valent Human Papillomavirus Vaccine in Belgium

**DOI:** 10.3389/fphar.2021.628434

**Published:** 2021-04-12

**Authors:** Steven Simoens, Andre Bento-Abreu, Barbara Merckx, Sophie Joubert, Steve Vermeersch, Andrew Pavelyev, Stefan Varga, Edith Morais

**Affiliations:** ^1^ KU Leuven Department of Pharmaceutical and Pharmacological Sciences, Leuven, Belgium; ^2^ Merck Sharp & Dohme, Brussels, Belgium; ^3^ Merck & Co., Inc., Kenilworth, NJ, United States; ^4^ HCL America, Inc., Sunnyvale, CA, United States; ^5^ Merck Sharp & Dohme, Lyon, France

**Keywords:** HPV, human papillomavirus, vaccine, cervical cancer, genital warts, cost-effectiveness, Belgium, 9-valent

## Abstract

**Background:** Routine human papillomavirus (HPV) immunization in Belgium is currently regionally managed, with school-aged girls receiving the 9-valent HPV (9vHPV) vaccine in Flanders and Wallonia-Brussels with a national catch-up program for females only. This study will assess whether expanding these programs to gender-neutral vaccination (GNV) with the 9vHPV vaccine is a cost-effective strategy in Belgium.

**Methods:** A validated HPV-type transmission dynamic model estimated the potential health and economic impact of regional vaccination programs, comparing GNV versus female-only vaccination (FOV) with the 9vHPV vaccine in individuals aged 11–12 years in Flanders, GNV with the 9vHPV vaccine versus FOV with the 2-valent HPV (2vHPV) vaccine in individuals aged 12–13 years in Wallonia-Brussels, and national catch-up GNV versus FOV with the 9vHPV vaccine for those aged 12–18 years. Vaccination coverage rates of 90, 50, and 50% in both males and females were used in the base cases for the three programs, respectively, and sensitivity analyses were conducted. All costs are from the third-party payer perspective, and outcome measures were reported over a 100-year time horizon.

**Results:** GNV with the 9vHPV vaccine was projected to decrease the cumulative incidence of HPV 6/11/16/18/31/33/45/52/58-related diseases relative to FOV in both Flanders and Wallonia-Brussels. Further reductions were also projected for catch-up GNV with the 9vHPV vaccine, including reductions of 6.8% (2,256 cases) for cervical cancer, 7.1% (386 cases) and 18.8% (2,784 cases) for head and neck cancer in females and males, respectively, and 30.3% (82,103 cases) and 44.6% (102,936 cases) for genital warts in females and males, respectively. As a result, a GNV strategy would lead to reductions in HPV-related deaths. Both regional and national catch-up GNV strategies were projected to reduce cumulative HPV-related disease costs and were estimated to be cost-effective compared with FOV with incremental cost-effectiveness ratios of €8,062, €4,179, and €6,127 per quality-adjusted life-years in the three programs, respectively. Sensitivity analyses were consistent with the base cases.

**Conclusions:** A GNV strategy with the 9vHPV vaccine can reduce the burden of HPV-related disease and is cost-effective compared with FOV for both regional vaccination programs and the national catch-up program in Belgium.

## Introduction

In 2007, the Superior Health Council of Belgium announced recommendations for human papillomavirus (HPV) vaccination for females aged 10 to 13 years and catch-up vaccination for females aged 14 to 26 years in addition to screening for the prevention of cervical cancer (Conseil Supérieur de la Santé, 2017; Thiry et al., 2019). HPV is a common sexually transmitted infection that can lead to the development of cervical, vulvar, and vaginal cancer in women, penile cancer in men, and genital warts, recurrent respiratory papillomatosis (RRP), and anal and head and neck cancers in both sexes (Schiffman and Castle, 2003; Forman et al., 2012; Giuliano et al., 2015; Fortes et al., 2017). HPV-related diseases impart a substantial burden on patients and their communities [The Belgian Health Care Knowledge Center (KCE), 2007]. In both Belgium and worldwide, there has been a trend for increasing HPV-related oropharyngeal cancer incidence, especially among males (incidences of 6.7 per 100,000 and 1.7 per 100,000 among males and females in Belgium, respectively (Bruni, 2017; de Martel et al., 2017). Furthermore, the estimated burden of cancers attributable to HPV 16/18 (excluding cervical cancer) in Europe has been shown to be higher in men than in women (32.2% vs. 19.4%, respectively, of new cases annually) and is driven primarily by head and neck cancers (26.4% vs. 5.3%, respectively) (Hartwig et al., 2012). Additionally, the likelihood of an HPV infection and rates of HPV-related disease (i.e., genital warts) and anal cancer are higher in male populations at high risk, such as men who have sex with men (MSM) (Nyitray et al., 2011; Anic et al., 2012; Frisch et al., 2003).

The importance of HPV vaccination is highlighted by the World Health Organization’s global strategy to achieve 90-70-90 targets (90% vaccine coverage, 70% cervical screening rates, 90% cervical disease treatment rates) by 2030 in an attempt to eliminate cervical cancer (World Health Organization, 2020). As a result, many countries have implemented cervical cancer screening programs and HPV immunization programs to reduce the incidence of cervical cancer and other HPV-related diseases, including the manifestation of genital warts (Bruni et al., 2016; Bruni, 2017; Murillo et al., 2008). In 2017, the Belgian Superior Health Council updated its recommendations to propose HPV vaccination for immunocompromized patients, girls and boys aged 9 to 14, and catch-up vaccination for women and men aged 15 to 26 years old (Thiry et al., 2019)].

**FIGURE 1:**
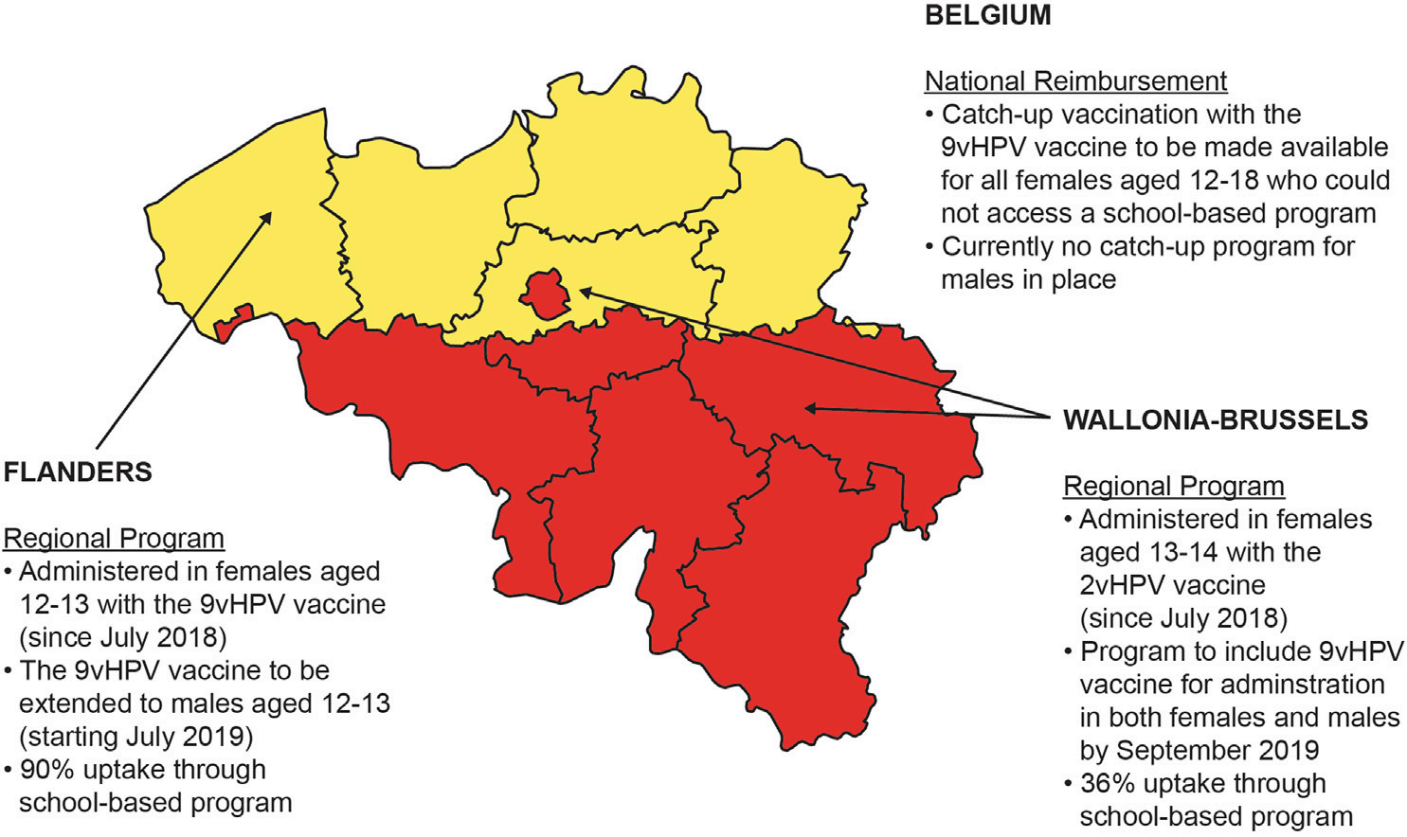
National reimbursement and regional programs for HPV vaccination in Belgium.

Vaccination in Belgium is made available through one of three options: reimbursement by the National Institute for Health and Disability Insurance, free of charge through regional community vaccination programs, or full price at pharmacies (Thiry et al., 2019). However, at the regional level, there are variations in the HPV vaccination recommendations and schedules (Figure 1). Belgium is divided into three geographical regions—the Flanders region (population of 6.1 million people), the Wallonia region (3.4 million people), and the capital region of Brussels (1 million people) (Institut National d’Etudes demographiques, 2009). Currently, routine HPV immunization programs in Belgium are managed by the community governments in Federation Wallonia-Brussels and Flanders with national reimbursement available only for catch-up HPV vaccination for girls aged 12 to 18 who did not have access to vaccination through a regional vaccination program (Thiry et al., 2019). HPV immunization programs for females were initiated in both the Flanders and Wallonia-Brussels regions of Belgium in 2010 and 2011, respectively (Thiry et al., 2019).

Two HPV vaccines are currently available as part of the Belgium national reimbursement program: the 2-valent HPV (2vHPV; targeting HPV types 16/18) and the 9-valent HPV (9vHPV; targeting HPV 6/11/16/18/31/33/45/52/58) vaccines (Agentschap Zorg and Gezondheid, 2018). The 9vHPV vaccine provides the broadest coverage, targeting HPV types 6/11, which are responsible for approximately 90% of genital warts cases globally (Garland et al., 2009); HPV types 16/18, which are responsible for approximately 73% of cervical cancer, 13% of vulvar cancer, 50% of vaginal cancers, 80% of anal cancer, 23% of penile cancer, and 19% to 36% of oropharyngeal cancer in Europe; and HPV types 31/33/45/52/58, which are responsible for additional smaller percentages of HPV-related cervical (16%), vulvar (10%), vaginal (15%), anal (3%), penile (12%) and oropharyngeal cancers (4%) in Europe (Nadiaye et al., 2014; Hartwig et al., 2017; de Sanjosé et al., 2018).

In Flanders, in 2010, the 4-valent HPV (4vHPV; targeting HPV 6/11/16/18) vaccine was made available for free in a 3-dose schedule through a regional vaccination program for females in the first year of secondary school (11–12 years old) (Geert, 2018). In 2014, the program introduced the 2vHPV vaccine in a 2-dose schedule, then in July 2018, the 9vHPV vaccine (administered in a 2-dose schedule) replaced the 2vHPV vaccine in the regional program. Vaccinating boys with the 9vHPV vaccine has been recommended since September 2017 and implemented since September 2019 (Vaccination Info Belgium, 2020a; Vaccination Info Belgium, 2020b). Vaccine uptake rates of approximately 90% in the Flanders region are for both two- and three-dose schedules of the HPV vaccine (Thiry et al., 2019).

In 2011, the 2vHPV vaccine was made available in the Wallonia-Brussels region through a free school-based program to females in the second year of secondary school (13–14-years-old; initially in a 3-dose schedule and since 2014 in a 2-dose schedule) (Bruni, 2017; Thiry et al., 2019; Vaccination Info Belgium, 2020a). Vaccination of boys with the 9vHPV vaccine has been also implemented since September 2019. Vaccine uptake in the Wallonia-Brussels region is lower than for Flanders, estimated at 29.2% and 36.1% from 1 to 2 and 5 to 6 years after the start of the program, respectively (Thiry et al., 2019).

Both Flanders and the Wallonia-Brussels regions share similar age-standardized incidence rates for cervical cancer (7.3 and 8.8 cases per 100,000, respectively). However, the Flanders region has a higher mortality rate from cervical cancer (1.9/100,000 person-years compared to 1.2/100,000 person-years in Wallonia) (Belgian Cancer Registry, 2020). In Belgium, the incidence of genital warts is high among men and women aged 23 to 30 years (145.82 and 220.11 per 100,000 person-years, respectively) (Dominiak-Felden et al., 2015). Furthermore, the incidence of head and neck cancer in Belgium is 3 times higher in males than in females (35.6 vs. 12.1 per 100,000 person-years) (Belgian Cancer Registry, 2017). However, limited data is available on the incidence of other HPV-related cancers in Belgium, with small retrospective studies reporting HPV was found in 22.3, 89.5, and 61.1% of oropharyngeal squamous cell carcinoma, penile intraepithelial neoplasia, and invasive penile cancers, respectively (Grisar et al., 2016; D’Hauwers et al., 2012).

There is a strong rationale for immunizing females and males against HPV. Although a declining trend in the incidence/prevalence of cervical cancer and other HPV-related diseases has been observed in females with the introduction of HPV vaccinations for girls in many countries, the burden of HPV-related diseases in men remains high, particularly HPV-related anal and oral cancers (Dunne et al., 2007; Giuliano et al., 2008; Global Burden of Disease Cancer Collaboration et al., 2015). Additionally, men rarely acquire immunity following natural HPV infection, and antibodies acquired after an infection do not appear to protect against subsequent HPV infections or HPV-related diseases (Beachler et al., 2018). Therefore, a GNV approach can directly protect males against HPV infection and HPV-related diseases and may further reduce HPV-related diseases in females. In recent years, several countries, most recently England, Germany, Netherlands, and France have introduced gender-neutral HPV vaccination to their national immunization programs to reduce the incidence of and burden associated with HPV-related diseases and cancers (Bruni, 2017; Takla et al., 2018; Kmietowicz, 2018).

Beginning in September 2019, both the Flanders and the Wallonia-Brussels regions in Belgium have switched to a gender-neutral HPV vaccination (GNV) program (Figure 1), joining in the international trend to combat HPV-related disease with HPV vaccination. In general, cost-effectiveness data are a key component in the decision-making process. A cost-effectiveness analysis of GNV with the 2vHPV, 4vHPV, and 9vHPV vaccines in the United Kingdom setting demonstrated that a GNV approach was less cost-effective than a female-only vaccination (FOV) approach, most likely because of the United Kingdom’s high vaccine uptake rates among females (2vHPV vaccine, 77–84%; 4vHPV vaccine, 85–88%) (Datta et al., 2019). A systematic review of cost-effectiveness studies evaluating GNV with the 9vHPV vaccine also indicated that the inclusion of adolescent boys into a vaccination program was cost-effective only if vaccine price and coverage were low (Ng et al., 2018). Recently, GNV with the 9vHPV vaccine was found to be cost-effective at high vaccine uptake rates (80%) in European tender-based settings (Qendri et al., 2020). Furthermore, the same HPV disease transmission dynamic model was adapted to the French setting, demonstrating that GNV with the 9vHPV vaccine had a significant impact on public health benefits and may be considered more cost-effective than an FOV strategy (Majed et al., 2021). In the Belgium setting, it is not known whether expansion of existing FOV programs to a GNV strategy with the 9vHPV vaccine will be cost-effective. Therefore, the objective of this study is to assess the potential health and economic impact of a GNV strategy with the 9vHPV vaccine vs. FOV in all current HPV vaccination programs in Belgium.

## Material and Methods

### Mathematical Model

#### Design

A previously validated and published HPV-type transmission dynamic model simulating the natural history of HPV infections and estimating the cost associated with HPV-related diseases in women has been extended to include all HPV-related diseases in both sexes (Elbasha et al., 2007; Elbasha & Dasbach, 2010; Boiron et al., 2016; Largeron et al., 2017; Mennini et al., 2017; Kind et al., 2020; Majed et al., 2021). HPV infection and disease state transitions, lifetime durations of infection-derived immunity, and unvaccinated compartments in this model have been described previously in detail (Elbasha et al., 2007). was adapted to the Belgium setting in order to evaluate the health and economic impact of GNV with the 9vHPV vaccine versus FOV with the 9vHPV in the Flanders region and the 2vHPV in the Wallonia-Brussels region. The analysis also evaluated the impact of catch-up 9vHPV GNV compared to 9vHPV FOV on the population of Belgium (base-case analysis).

#### Model Compartments

The age-structured mathematical model (Elbasha et al., 2007) accounts for herd immunity and comprises three connected modules: 1) a demographic variables model describing birth, aging, and death; 2) a behavioral model describing sexual activity and sexual mixing patterns; and 3) a disease variables model describing screening rates and HPV infection and related disease transmission. In this model, individuals enter the population at a gender-specific and sexual activity–specific rate with an assumed age of sexual debut of 12 years and move between successive age groups at an age- and gender-specific rate per year (Elbasha et al., 2007). These individuals exit the model upon death at an age- and gender-specific per capita death rate per year (Elbasha et al., 2007). Patients with cervical cancer have an additional age- and stage-dependent death rate, but it was assumed that those with other HPV-related diseases [i.e., CIN, vaginal/vulvar cancers, vaginal intraepithelial neoplasia (VaIN), vulvar intraepithelial neoplasia (VIN), anal cancer, head and neck cancer, penile cancer, RRP, or genital warts] do not face an additional risk of death (Elbasha et al., 2007). The age-structured model also simulates HPV transmission and the occurrence of CIN, cervical cancer, external genital warts, and other HPV-related disease. The acquisition of an infection and progression of individuals from infection to disease follow a similar natural history structure, as assumed in previous models for HPV 16/18 (Dasbach et al., 2006). We also incorporated HPV 6/11/31/33/45/52/58 infections and associated diseases and grouped infections into HPV 16/18, HPV 6/11, or HPV 6/11/16/18/31/33/45/52/58. The population was divided into distinct epidemiological categories according to the individual’s status with respect to infection, disease, screening, and treatment (Elbasha et al., 2007). Further details of the model structure and assumptions have been published (Elbasha et al., 2007).

### Epidemiological Model Parameters

Model input variables of the epidemiological model are divided into demographics, sexual behavior, disease and treatment patterns, and screening. A detailed summary of these parameter groups is provided in Supplementary Table S1 along with the data sources. When available, local Belgium-specific data were used.

#### Demographics

Population figures and annual all-cause mortality rates for the general population by sex and age were retrieved from local Belgium-specific data (Supplementary Table S1). An overall Belgian population of 11,322,088 individuals was included in the model, which comprised 5,754,083 females and 5,568,005 males (STATBEL, 2019). Annual all-cause mortality by age cohorts varied from 0.00075% among adolescent/adult males (15–44 years) to 0.09144% (≥75 years) among the elderly male population, and from 0.00035% among adolescent/adult females (15–44 years) to 0.06844% (≥75 years) among the elderly female population (La Belgique en Chiffres, 2019).

#### Sexual Behavior

Data from France were utilized for sexual behavior variables with the exceptions mentioned below (Supplementary Table S1). The annual mean number of sexual partners for age groups between 13 and ≥70 years ranged from 0.0499 (13–14 years) to 1.50 (20–24 years) among males, and from 0.0778 (13–14 years) to 1.20 (18–29 years) among females (Bajos & Boson et al., 2008); United States data were used for males aged 13–17 years and ≥70 years (Elbasha and Dasbach, 2010). The proportions of the population at low (mean number of sexual partners per year of 0–1), medium (2–4 sexual partners per year), and high (5 or more sexual partners per year) sexual activity risk were reported among males (87.71, 9.66, and 2.63%, respectively) and females (92.85, 6.38, and 0.77%, respectively) (Bajos and Boson et al., 2008). The mean numbers of sexual partners per year by low, medium, and high sexual activity risk group were also reported among males (0.86, 2.65, and 8.04, respectively) and females (0.84, 2.58, and 7.93, respectively) (Bajos and Boson et al., 2008).

The degree of sexual mixing among members of different age cohorts and sexual activity groups (0 representing no mixing, and 1 representing a maximal mixing) were extracted from United States-specific data, (Elbasha and Dasbach, 2010) and was adjusted during the calibration process. Mixing between debut and cessation, and after cessation were 0.40 and 0.10, respectively (Elbasha and Dasbach, 2010).

#### Disease and Treatment Patterns

Model input variables to simulate the natural history of the disease as well as disease and treatment patterns utilized local Belgium-specific data when available or estimated through model calibration (Supplementary Table S1).

Belgium-specific data on the incidence of HPV-related diseases were retrieved from previously published data and the Belgian Cancer Registry (Dominiak-Felden et al., 2015; Belgian Cancer Registry, 2020). Female incidence rates (per 100,000 person-years) for cervical cancer, vaginal cancer, vulvar cancer, anal cancer, head and neck cancer, and genital warts in Belgium were 11.12, 0.73, 3.80, 2.27, 10.78, and 113.62, respectively (Dominiak-Felden et al., 2015; Belgian Cancer Registry, 2020). Male incidence rates for anal cancer, head and neck cancer, penile cancer, and genital warts in Belgium were 1.43, 31.10, 1.70, and 71.13, respectively (Belgian Cancer Registry, 2020; Dominiak-Felden et al., 2015). Incidence rates for the various HPV-related cancers and genital warts were estimated by applying the proportion of diseases attributable to all HPV types, and types in the 4vHPV and 9vHPV vaccines in Europe (Hartwig et al., 2017) with the exception of the proportion of HPV-related oropharynx cancers in Belgium, obtained from clinical experts (S. Nuyts, personal communication, February 21, 2019 and M. Remacle, personal communication, February 27, 2019).

Annual mortality rates for each HPV-related cancer, stratified by age and stage (local, regional, and distant disease), are summarized in Table 1. These were obtained by combining the data from two sources: survival data from the European Cancer Registry (EUROCARE-5) database (2000 to 2007) and Cancer Survival data from the Belgian Cancer Registry (2004 to 2008) (Belgian Cancer Registry, 2020).

**TABLE 1:** Summary table on cancer mortality.

Cancer type	Age group (years)	Annual mortality rate		
		Local cancer^a^	Regional cancer^a^	Distant cancer^a^
Cervical cancer	15–44	0.009	0.030	0.075
	45–54	0.020	0.063	0.157
	55–64	0.029	0.094	0.236
	65–74	0.035	0.112	0.283
	75+	0.077	0.245	0.617
Vaginal cancer	15–44	0.033	0.057	0.102
	45–54	0.018	0.031	0.055
	55–64	0.048	0.083	0.148
	65–74	0.073	0.125	0.223
	75+	0.122	0.211	0.376
Vulvar cancer	15–44	0.026	0.057	0.124
	45–54	0.014	0.031	0.067
	55–64	0.038	0.083	0.179
	65–74	0.058	0.125	0.271
	75+	0.097	0.211	0.456
Anal cancer (females)	15–44	0.028	0.062	0.106
	45–54	0.033	0.074	0.127
	55–64	0.032	0.072	0.123
	65–74	0.043	0.095	0.163
	75+	0.084	0.187	0.321
Anal cancer (males)	15–44	0.031	0.068	0.118
	45–54	0.030	0.067	0.115
	55–64	0.040	0.090	0.154
	65–74	0.051	0.114	0.196
	75+	0.090	0.199	0.342
Penile cancer	15–44	0.008	0.037	0.082
	45–54	0.014	0.066	0.145
	55–64	0.013	0.061	0.134
	65–74	0.021	0.100	0.220
	75+	0.042	0.200	0.441
Head and neck cancer (females)	15–44	0.053	0.084	0.146
	45–54	0.069	0.109	0.191
	55–64	0.076	0.120	0.209
	65–74	0.088	0.139	0.242
	75+	0.155	0.244	0.427
Head and neck cancer (males)	15–44	0.066	0.132	0.235
	45–54	0.078	0.156	0.277
	55–64	0.088	0.175	0.311
	65–74	0.101	0.202	0.358
	75+	0.152	0.302	0.537

^a^ Disease stages can be related to the traditional TNM classification system as follows: “local disease” corresponds to stages I and II TNM classification (localized primary tumor); “regional disease” corresponds to stage III TNM classification system (metastasis to regional lymph nodes); “distant disease” corresponds to stage IV TNM classification system (distant metastatic disease), TNM, tumor-node-metastasis.

The proportions of the populations with HPV-related diseases (including cervical cancer [including the precancerous lesions cervical intraepithelial neoplasia {CIN 1/2/3} and carcinoma *in situ* {CIS}], vaginal cancer [including the precancerous lesions VaIN 1/2/3], vulvar cancer [including the precancerous lesions VIN 1/2/3], anal cancer, genital warts, and head and neck cancer for females; and anal cancer, genital warts, penile cancer, head and neck cancer for males) recognizing their symptoms and seeking treatment, and the proportions who were treated were estimated through the model calibration. Similarly, the proportions of females and males who were treated for genital warts were estimated through the model calibration.

#### Screening

The proportion of females receiving a follow-up cervical cancer screening test after abnormal PAP smear diagnosis (90.21%), and the proportion of females receiving gynecological cancer screening tests at least once every 3 years (53.70%) were calculated based on data from the Belgian Healthcare Knowledge Center (KCE) and a 2007 expert Belgium survey (Supplementary Table S1). Cervical cancer screening adherence rates for females stratified by age were also calculated based on KCE data. In terms of diagnostic performance for cervical screening, the sensitivity and specificity of colposcopy were 96 and 48%, respectively, whereas the specificity of the PAP screening test was 94% [The Belgian Health Care Knowledge Center (KCE), 2007]. In addition, the sensitivity of PAP screening for CIN was 63, 61, and 61% for CIN 1, CIN 2, and CIN 3, respectively (Arbyn et al., 2015).

#### Vaccine Properties

The prophylactic efficacy or the degree of protection offered by the vaccine was based on clinical trial data (Ault, 2007; Joura et al., 2007; Garland et al., 2007; Giuliano et al., 2011; Joura et al., 2015; Palefsky et al., 2011). The duration of protection against HPV genotypes 6/11/16/18/31/33/45/52/58 was assumed to be lifelong (except for the 20-year duration of protection in the sensitivity analysis) and herd immunity was assumed. Table 2 summarizes the vaccine efficacy parameters related to the protection against both transient and persistent infections.

**TABLE 2:** Summary table on vaccine characteristics (Joura et al., 2007; Garland et al., 2007; Guiliano et al., 2011; Joura et al., 2015; Palefsky et al., 2011; Ault, 2007).

Vaccine efficacy against HPV infection	Vaccine assumptions (percentage of exposed people avoiding the infection)				
	HPV type 6	HPV type 11	HPV type 16	HPV type 18	HPV types 31/33/45/52/58
Protection against transient infection^a^					
Anal, head and neck, penile, vaginal, and vulvar infection					
Male^b^	—	—	41.1	62.1	—
Female^c^	—	—	76.0	96.3	—
Cervical infection					
Male^b^	—	—	41.1	62.1	41.1
Female^c^	—	—	76.0	96.3	76.0
Infection associated with genital warts and RRP					
Male^b^	49.0	57.0	—	—	—
Female^c^	76.1	76.1	—	—	—
Protection against persistent infection					
Anal and head and neck infection^d^					
Male^b^	—	—	78.7	96.0	—
Female^c^	—	—	98.8	98.4	—
Cervical infection (female only)	—	—	98.8	98.4	98.8
Penile infection (male only)^d^	—	—	78.7	96.0	—
Vaginal and vulvar infection (female only)	—	—	98.8	98.4	—
Protection against individual HPV-related diseases					
CIN (female only)	100.0	100.0	97.9	100.0	97.9
VIN (female only)	—	—	100.0	100.0	—
VaIN (female only)	—	—	100.0	100.0	—
Genital warts					
Male^b^	84.3	90.9	—	—	—
Female^c^	98.9	100.0	—	—	—

^a^ Efficacy for 1 or 2 doses of vaccine assumed to be 0.

^b^ Preventing male genital infections through male vaccination is assumed to prevent transmission of genital infections to females.

^c^ Preventing female genital infections through vaccination is assumed to prevent transmission of genital infections to males.

^d^ The efficacy against anal, head and neck, penile, and RRP diseases is conferred through protection against infection only.

CIN, cervical intraepithelial neoplasia; HPV, human papillomavirus; RRP, recurrent respiratory papillomatosis; VaIN, vaginal intraepithelial neoplasia; VIN, vulvar intraepithelial neoplasia.

### Economic Model Parameters

The inputs for the economic model included vaccine strategy, vaccine properties, cost parameters, and health-related quality of life.

#### Vaccine Strategies and Coverage Rates

The vaccination schedule was assessed according to the product label, i.e., 2-dose schedule for children under 15 years and 3-dose schedule for children between 15 and 18 years old. Our base-case analyses were conducted according to three different scenarios: 1) in the Flanders region, strategy of GNV vs. FOV with 9vHPV vaccine administered as two doses in ages 11 to 12 years old; 2) in the Wallonia-Brussels region, strategy of GNV with the 9vHPV vaccine vs. FOV with the 2vHPV vaccine, both administered as two doses in ages 12 to 13 years old; and 3) in the national catch-up program, strategy of GNV vs FOV with 9vHPV vaccine administered as two doses in ages 12 to 14 and 3 doses in ages 15 to 18 years old.

GNV versus FOV with the 9vHPV vaccine in children ages 11 to 12 years was assessed at a 90% vaccine coverage rate (VCR) in Flanders, reflecting the high coverage in this region (Tjalma et al., 2018). GNV with the 9vHPV vaccine versus FOV with the 2vHPV vaccine in children aged 12 to 13 years was assessed at a VCR of 50% in the Wallonia-Brussels region. The national catch-up GNV versus catch-up FOV with the 9vHPV vaccine was assessed at a predicted VCR of 50% in males and females. This VCR is a 10- to 15-year projection allowing for the regional (school) program to provide protection for the entire population (catch-up cohort) analyzed in the base case and sensitivity scenarios. Catch-up vaccination was modeled in children aged 12 to 18 years to reflect the current national immunization program.

#### Cost Parameters

Cost parameters used in the model included cost per episode of care, cost of vaccination, and cost of screening and diagnostic tests; costs were reported in 2017 euros (€). A detailed summary of all costs used in the model is provided in Table 3.

**TABLE 3:** Summary table on cost parameters for HPV-related diseases (third-party payer perspective; 2017 €).

Parameter	Cost (2017 €)	Data source
**Cost of vaccination**		
Per dose		Belgium National institute for health and disability (INAMI) (2019)
9vHPV vaccine	123.97	
2vHPV vaccine	55.00	
Administration per dose		
9vHPV vaccine	NA	
2vHPV vaccine	NA	
Total cost of single dose of vaccine plus administration		
9vHPV vaccine	123.97	
2vHPV vaccine	55.00	
**Cost per episode-of-care** ^a^		
CIN 1 (female only)	267.06	Annemans et al. (2008)
CIN 2 (female only)	355.55	
CIN 3 and CIS (female only)	458.75	
Cervical cancer, local disease^b^ (female only)	11,484	Tsakeu et al. (2016)
Cervical cancer, regional disease^b^ (female only)	15,330	
Cervical cancer, distant disease^b^ (female only)	18,608	
VaIN 2 (female only)	711	Assumed twice as expensive as CIN costs
VaIN 3 and CIS (female only)	917	
Vaginal cancer, local disease^b^ (female only)	12,876	Tsakeu et al. (2016)
Vaginal cancer, regional disease^b^ (female only)	12,873	
Vaginal cancer, distant disease^b^ (female only)	26,505	
Vulvar cancer, local disease^b^ (female only)	15,076	
Vulvar cancer, regional disease^b^ (female only)	21,145	
Vulvar cancer, distant disease^b^ (female only)	28,468	
Penile cancer, local disease^b^ (male only)	15,312	
Penile cancer, regional disease^b^ (male only)	26,270	
Penile cancer, distant disease^b^ (male only)	28,694	
Anal cancer, local disease^b^		
Female	19,447	
Male	16,537	
Anal cancer, regional disease^b^		
Female	28,687	
Male	28,823	
Anal cancer, distant disease^b^		
Female	17,038	
Male	20,156	
Head and neck cancer, local disease^b^		
Female	13,516	
Male	16,644	
Head and neck cancer, regional disease^b^		
Female	21,818	
Male	21,121	
Head and neck cancer, distant disease^b^		
Female	17,271	
Male	22,907	
Genital warts		Annemans et al. (2008)
Female	391	
Male	385	
RRP^c^		Hughes et al. (2011)
Female	22,507	
Male	22,507	
**Screening and diagnostic tests** ^d^ **(female only)**		
Screening (PAP smear and office visit)	41	Belgium National institute for health and disability (INAMI) (2019)
Colposcopy	14	Arbyn et al. (2015)
Biopsy	74	

^a^ Inflation of 3.982% (2015–2017) was applied (obtained from: statbel.fgov.be).

^b^ Disease stages can be related to the traditional Tumor-Node-Metastasis (TNM) classification system as follows: “local disease” corresponds to stages I and II TNM classification (localized primary tumor); “regional disease” corresponds to stage III TNM classification system (metastasis to regional lymph nodes); “distant disease” corresponds to stage IV TNM classification system (distant metastatic disease).

^c^ Assumed that 3% was at the charge of the patient.

^d^ For cervical and vaginal cancers only.

2vHPV, 2-valent human papillomavirus vaccine; 9vHPV, 9-valent human papillomavirus vaccine; CIN, cervical intraepithelial neoplasia; HPV, human papillomavirus; NA, not applicable; RRP, recurrent respiratory papillomatosis; VaIN, vaginal intraepithelial neoplasia; VIN, vulvar intraepithelial neoplasia.

##### Cost of Vaccination

The base model adopted the public prices of vaccines in Belgium (€55 and €123.97 for the 2vHPV and 9vHPV vaccines, respectively).

##### Cost Per Episode of Care

The estimated costs per episode of care of each HPV-related disease, defined as the cost of management from diagnosis to resolution of the case, are summarized in Table 3. Costs were retrieved from Annemans et al. (Annemans et al., 2008) and real-world data from the IMS Health Belgian Hospital Disease Database (Hughes et al., 2011; Tsakeu et al., 2016).

##### Cost of Screening and Diagnostic Tests

For cervical and vaginal cancers, a 2016 report by the Belgian National Institute for Health and Disability Insurance (INAMI) was used to extract the cost associated with screening (PAP smear) and related office visits, whereas colposcopy and biopsy costs were retrieved from a 2015 update on HPV primary screening by the KCE (Arbyn et al., 2015). Costs for screening by PAP smear were set at €41, colposcopy at €14, and biopsy at €74.

##### Perspective

All costs are from the perspective of the third-party payer (i.e., only direct costs were included in the analysis).

##### Discount Rates

Discount rates of 1.5% for quality-adjusted life years (QALYs) and 3% for costs were applied to the model. These discount rates were in accordance with the Belgian KCE Guidelines for pharmaco-economic evaluation (Cleemput et al., 2008b).

#### Health-Related Quality of Life

The health utility values for the Belgian general population and for those with HPV-related diseases were derived from several sources. Health utilities for the Belgian general population by age and sex were based on data from the United Kingdom (Cleemput et al., 2004; Szende et al., 2014) and are summarized in Table 4. Disease-related health utilities for HPV-related diseases by sex and disease stage in the Belgian general population were estimated using United Kingdom- and United States-based data; these are also summarized in Table 4 (Szende and Williams et al., 2004; Hu & Goldie, 2008; Chadha et al., 2010; Sullivan et al., 2011; Dominiak-Felden et al., 2013).

**TABLE 4:** Summary table on health utilities for individuals without HPV disease (by age) and for individuals with HPV-related diseases (by disease stage).

	Females	Males	
**Individuals without HPV disease**			
**Age group (years)**			
<15	0.849	0.850	Szende and Williams (2004), Cleemput et al. (2004)
5–29	0.849	0.850	
30–39	0.816	0.837	
40–49	0.827	0.820	
50–59	0.801	0.798	
60–69	0.800	0.773	
70–79	0.733	0.714	
80+	0.686	0.730	
**Individuals with HPV-related disease**			
CIN 1/2/3	0.822	NA	Hu and Goldie (2008), Sullivan et al. (2011)
CIS (cervical)	0.822	NA	
Cervical cancer, local	0.822	NA	
Cervical cancer, regional	0.732	NA	
Cervical cancer, distant	0.542	NA	
Cervical cancer, cancer survivor	0.822	NA	
VaIN 2/3	0.822	NA	
CIS (Vaginal)	0.822	NA	
Vaginal cancer, local	0.822	NA	
Vaginal cancer, regional	0.732	NA	
Vaginal cancer, distant	0.542	NA	
Vaginal cancer, cancer survivor	0.822	NA	
Vulvar cancer, local	0.822	NA	
Vulvar cancer, regional	0.732	NA	
Vulvar cancer, distant	0.542	NA	
Vulvar cancer, cancer survivor	0.822	NA	
Penile cancer, local	NA	0.751	
Penile cancer, regional	NA	0.661	
Penile cancer, distant	NA	0.471	
Penile cancer, cancer survivor	NA	0.751	
Anal cancer, local	0.645	0.645	
Anal cancer, regional	0.555	0.555	
Anal cancer, distant	0.365	0.365	
Anal cancer, cancer survivor	0.645	0.645	
Head and neck cancer, local	0.756	0.756	
Head and neck cancer, regional	0.666	0.666	
Head and neck cancer, distant	0.476	0.476	
Head and neck cancer, cancer survivor	0.756	0.756	
Genital warts	0.900	0.900	Dominiak-Felden et al. (2013)
Recurrent respiratory papillomatosis	0.760	0.760	Chadha et al. (2010)

CIN, cervical intraepithelial neoplasia; CIS, carcinoma *in situ*; HPV, human papillomavirus; NA, not applicable; VaIN, vaginal intraepithelial neoplasia.

### Outcome Parameters

#### Outcome Measures

The model was used to estimate the following health outcome parameters: cumulative incidence of HPV-related diseases associated with HPV types targeted by the 9vHPV vaccine (6/11/16/18/31/33/45/52/58); number of prevented cases of HPV-related diseases (expressed as the cumulative reduction in HPV 6/11/16/18/31/33/45/52/58-related incident cases); number of prevented HPV-related deaths (expressed as the cumulative reduction in HPV 6/11/16/18/31/33/45/52/58-related incident cases). The model was also used to estimate the following economic outcome parameters: cumulative HPV-related disease health care costs; QALYs of the model population; and the incremental cost-effectiveness ratios (ICERs), which are calculated with the quotient: Incremental vaccination costs/Incremental QALYs. Model calculations were performed using the mathematical software package Mathematica®, Version 10.4 (Wolfram Research, Champaign, IL).

#### Time Horizon

Outcome measures were reported over a time horizon of 100 years because this was an appropriate time frame from which the system approached steady state and most benefits and costs of vaccination could be realized.

### Model Calibration and Validation

Validation of the original model has been previously addressed (Elbasha et al., 2007; Elbasha and Dasbach, 2010). Consultation with experts on assumptions regarding the natural history of HPV infection and disease and vaccine characteristics was undertaken to establish face validity of the model. To confirm the predictive validity of the model, it was shown that model predictions of HPV prevalence and disease outcomes generally fell within the range of values reported in the literature. To assess the convergent validity of the original model, its estimates were compared with those of several previously published studies, revealing consistent predictions of population-level effectiveness and herd effects (Brisson et al., 2016). Results from the Elbasha model were consistent with those of other dynamic transmission models (Brisson et al., 2016).

The incidence rates for genital warts; CIN 1/2/3; and cervical, vaginal, vulvar, anal, penile, and head and neck cancers were calibrated.

All HPV-related diseases were considered, in accordance with the Belgian Superior Health Council recommendations and other health technology assessment agencies considering HPV vaccination (Takla et al., 2018; Conseil Supérieur de la Santé, 2017; Favaretti et al., 2017; Joint committee on Vaccination and Immunization, 2018; Health Information and Quality Authority, 2018; KCE, 2019). Data were validated by national and international experts during an advisory board and conducted in agreement with the recent KCE report on HPV [The Belgian Health Care Knowledge Center (KCE), 2007].

### Sensitivity Analyses

Deterministic one-way sensitivity analyses were conducted to assess the sensitivity of ICER values to variables that have been shown to be impactful to cost effectiveness. One variable is the VCR, which were assessed at different levels (5–95% for male and female) (De La Fuente et al., 2019; Largeron et al., 2017). The lower VCR of 5% in the national catch-up program is a closer representation of the current eligible population, when including all the school-aged males and some females who may not receive vaccination through the school program. Sensitivity analyses also assessed a genital warts incidence rate of 76 per 100,000 (low) and 250 per 100,000 (high); lower (−20%) and higher (+20%) incidence of HPV-related cancers; label-only indications for the 9vHPV vaccine (genital warts, precancerous lesions [CIN 1/2/3, VIN 2/3, VaIN 2/3, anal intraepithelial neoplasia 1/2/3], cervical, vulvar, vaginal, and anal cancers for females; and genital warts and anal cancer for males); duration of protection of 20 years; vaccine price discount of ±10%; utilities (±10%); GNV with 9vHPV vs. 2vHPV; and FOV with 9vHPV versus FOV with 2vHPV.

## Results

### Regional Vaccination Program in Flanders

Projected onto the population of Flanders, 9vHPV vaccination in both males and females demonstrated greater cumulative reductions in HPV 6/11/16/18/31/33/45/52/58-related diseases relative to FOV over a 100-year period (Table 5). GNV with the 9vHPV vaccine is projected to decrease the cumulative incidence of anal, penile, and head and neck cancers in males by 429 (16.5%), 490 (28.6%), and 2,346 (19.1%) cases, respectively. Notably, GNV is also projected to benefit females, by reducing incidence of cervical cancer by 436 cases (2.7%), CIN 1 by 1,033 cases (5.3%), CIN 2/3 by 1,565 cases (5.0%), and HPV 6/11-related CIN 1 by 3,564 cases (35.5%). In addition, a cumulative incidence of genital warts was projected to decrease by 53,538 (60.1%) and 30,288 (35.9%) cases in males and females, respectively.

**TABLE 5:** Cumulative reduction in HPV 6/11/16/18/31/33/45/52/58-related disease incidence for gender-neutral vaccination with the 9vHPV vaccine compared with 9vHPV vaccination in females only in the Flanders Region, 2vHPV Vaccination in Females Only in the Wallonia-Brussels regions, and 9vHPV Vaccination in Females Only nationwide in Belgium over 100 years.

	Cumulative reduction in HPV 6/11/16/18/31/33/45/52/58-related incident cases over 100 years, n (%)		
	Flanders region (GNV 9vHPV vs. FOV 9vHPV)	Wallonia-Brussels region (GNV 9vHPV vs. FOV 2vHPV)	National catch-up (GNV 9vHPV vs. FOV 9vHPV)
**Cervical**			
Cancer	436 (2.7)	2,025 (16.4)	2,256 (6.8)
CIN 1	1,033 (5.3)	7,601 (32.3)	6,874 (13.8)
CIN 2/3	1,565 (5.0)	10,522 (29.3)	10,301 (13.0)
**Vaginal**			
Cancer	4 (2.3)	7 (5.5)	22 (5.9)
VaIN 2/3	0 (0.0)	0 (0.0)	0 (0.0)
**Vulvar**									
Cancer	6 (2.3)	10 (5.7)	31 (6.1)
**Genital warts and HPV 6/11-related CIN 1**			
Genital warts (female)	30,288 (35.9)	119,708 (65.2)	82,103 (30.3)
Genital warts (male)	53,538 (60.1)	73,421 (63.1)	102,936 (44.6)
CIN 1	3,564 (35.5)	12,117 (61.6)	9,018 (28.6)
**Anal**									
Cancer (female)	132 (3.7)	182 (7.7)	553 (7.4)
Cancer (male)	429 (16.5)	294 (17.7)	953 (17.8)
**Head and neck**									
Cancer (female)	133 (3.6)	155 (6.4)	386 (7.1)
Cancer (male)	2,346 (19.1)	1,391 (18.0)	2,784 (18.8)
**Penile**									
Cancer	490 (28.6)	206 (20.8)	461 (22.1)
**RRP**									
Female	97 (38.4)	343 (65.1)	244 (31.0)
Male	137 (52.1)	270 (63.7)	284 (38.4)

2vHPV, 2-valent human papillomavirus vaccine; 9vHPV, 9-valent human papillomavirus vaccine; CIN, cervical intraepithelial neoplasia; FOV, female-only vaccination; GNV, gender-neutral vaccination; HPV, human papillomavirus; RRP, recurrent respiratory papillomatosis; VaIN, vaginal intraepithelial neoplasia.

As a result of reductions in the incidence of HPV-related diseases, the model predicts cumulative reduction in HPV 6/11/16/18/31/33/45/52/58-related deaths over 100 years (Table 6), with the greatest reduction in deaths from head and neck cancer (1,175 for males and 43 for females, respectively), cervical cancer (127), anal cancer (81 for males and 40 for females), and penile cancer (67).

**TABLE 6:** Cumulative reduction in HPV 6/11/16/18/31/33/45/52/58-related deaths over 100 years with 9vHPV vaccination in females and males compared with female-only vaccination.

	Cumulative reduction in HPV 6/11/16/18/31/33/45/52/58-related deaths, n (%)		
	Flanders (GNV 9vHPV vs. FOV 9vHPV)	Wallonia-Brussels (GNV 9vHPV vs. FOV 2vHPV)	National catch-up (GNV 9vHPV vs. FOV 9vHPV)
Cervical cancer	127 (2.5)	525 (14.1)	594 (5.8)
Vaginal cancer	1 (2.1)	1 (4.7)	5 (5.0)
Vulvar cancer	2 (2.1)	2 (5.0)	8 (5.2)
Anal cancer (female)	40 (3.3)	53 (6.8)	160 (6.5)
Anal cancer (male)	81 (14.8)	56 (15.9)	176 (15.9)
Head and neck cancer (female)	43 (3.3)	48 (5.7)	120 (6.4)
Head and neck cancer (male)	1,175 (18.0)	688 (17.0)	1,379 (17.8)
Penile cancer	67 (25.2)	29 (18.5)	63 (19.7)
RRP (female)	4 (31.5)	15 (61.5)	11 (27.7)
RRP (male)	6 (44.5)	12 (60.2)	13 (34.8)

2vHPV, 2-valent human papillomavirus vaccine; 9vHPV, 9-valent human papillomavirus vaccine; FOV, female-only vaccination; GNV, gender-neutral vaccination; HPV, human papillomavirus; RRP, recurrent respiratory papillomatosis.

Upon evaluating the economic impact of the associated projected reductions in incidence associated with GNV relative to FOV with the 9vHPV vaccine, the Flanders region is expected to see a 5.9% reduction (€21,851,508) in cumulative HPV 6/11/16/18/31/33/45/52/58-related disease costs (Table 7); 54.1 and 44.4% of the reduction in cumulative HPV 6/11/16/18/31/33/45/52/58-related disease costs were attributable to HPV 16/18- and HPV 6/11-related disease costs, respectively.

**TABLE 7:** Estimated reductions in cumulative HPV-related disease costs over 100 years with 9vHPV vaccination in females and males compared with female-only vaccination.

	Cumulative HPV 6/11/16/18/31/33/45/52/58-related disease costs over 100 years (2017 €)								
	Flanders			Wallonia-Brussels			National catch-up		
	FOV 9vHPV	GNV 9vHPV	% Reduction with GNV	FOV 2vHPV	GNV 9vHPV	% Reduction with GNV	FOV 9vHPV	GNV 9vHPV	% Reduction with GNV
**Cervical**									
Cancer	121,463,664	120,090,179	1.1	75,110,163	70,838,451	5.7	221,068,799	215,727,358	2.4
CIN 1	646,876	627,818	2.9	511,252	425,167	16.8	1,245,220	1,166,577	6.3
CIN 2/3	5,582,165	5,432,369	2.7	4,208,443	3,673,786	12.7	10,762,979	10,149,886	5.7
**Vaginal**									
Cancer	1,635,803	1,621,481	0.9	935,144	918,495	1.8	2,923,035	2,866,547	1.9
VaIN 2/3	0	0	0	0	0	0	0	0	0
**Vulvar**									
Cancer	2,669,528	2,645,119	0.9	1,534,649	1,506,204	1.9	4,780,932	4,684,450	2.0
**Genital warts and HPV 6/11-related CIN 1**									
CIN 1	244,805	205,711	16.0	261,698	150,875	42.3	529,205	438,059	17.2
Genital warts (male)	15,056,393	10,019,758	33.5	14,357,243	7,638,673	46.8	31,993,851	22,026,928	31.2
Genital warts (female)	18,983,387	15,698,664	17.3	23,020,233	11,888,494	48.4	42,129,959	33,846,351	19.7
**Anal**									
Cancer (male)	21,298,329	20,035,436	5.9	12,437,627	11,636,169	6.4	40,377,601	37,660,056	6.7
Cancer (female)	30,871,133	30,498,619	1.2	18,513,400	18,064,295	2.4	58,346,553	56,959,678	2.4
**Head and neck**									
Cancer (male)	106,565,385	99,134,494	7.0	60,876,996	56,765,509	6.8	118,112,189	109,546,861	7.3
Cancer (female)	27,060,966	26,732,398	1.2	15,842,344	15,519,383	2.0	35,862,224	35,033,232	2.3
**Penile**									
Cancer	11,495,546	10,322,974	10.2	6,597,901	6,083,218	7.8	13,692,130	12,518,954	8.6
**RRP**	6,034,979	4,701,432	22.1	6,861,054	3,579,656	47.8	13,224,158	10,215,656	22.8
**Total disease costs**	369,608,960	347,757,452	5.9	241,068,147	208,688,374	13.4	595,048,835	552,840,593	7.1

2vHPV, 2-valent human papillomavirus vaccine; 9vHPV, 9-valent human papillomavirus vaccine; CIN, cervical intraepithelial neoplasia; FOV, female-only vaccination; GNV, gender-neutral vaccination; HPV, human papillomavirus; RRP, recurrent respiratory papillomatosis.

**FIGURE 2:**
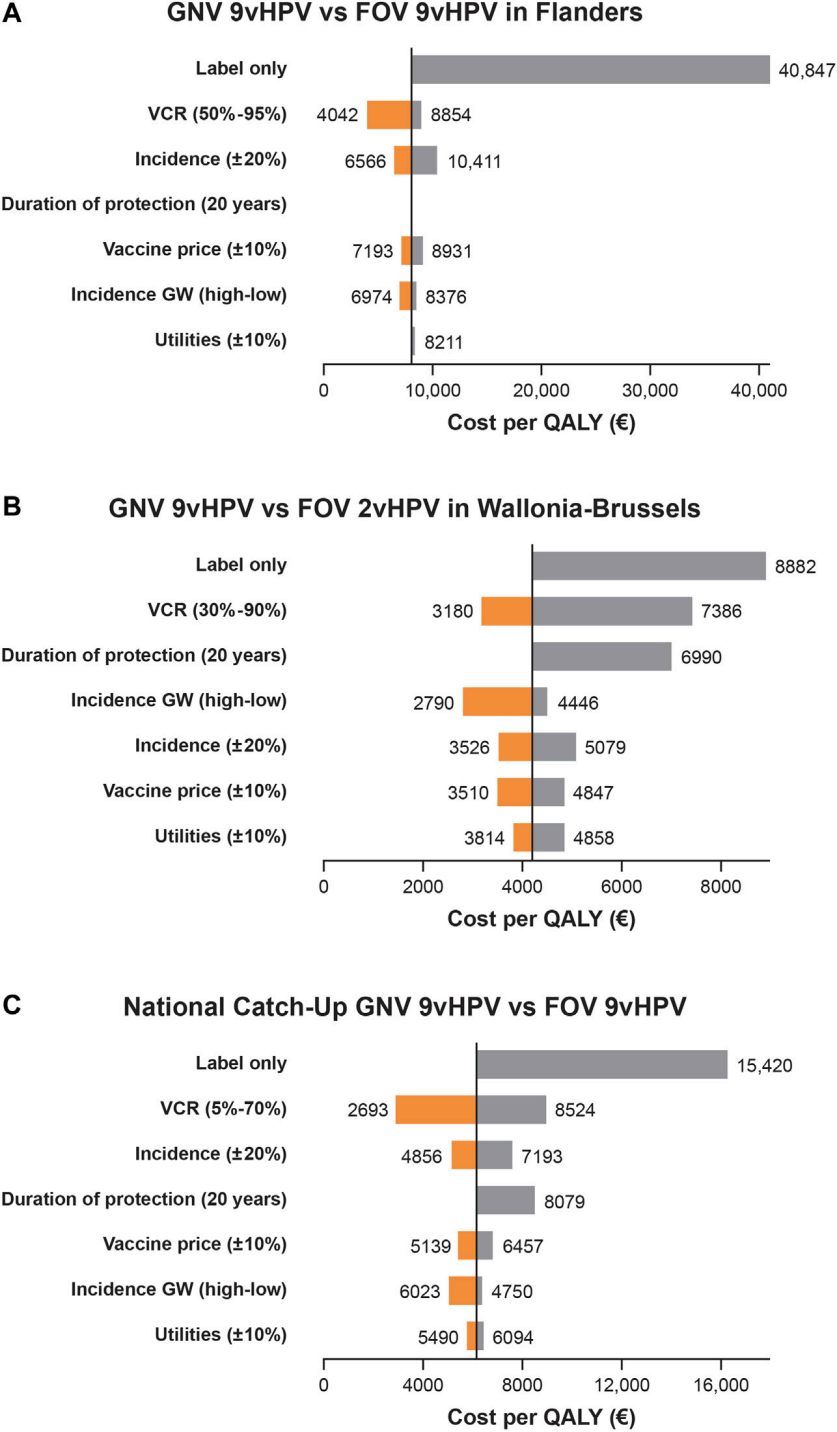
Univariate sensitivity analyses of the base case for the incremental cost-effectiveness ratio (ICER; cost per QALY) of gender-neutral vaccination (GNV) with the 9vHPV vaccine in the Flanders Region of Belgium versus female-only vaccination [FOV] with the 9vHPV Vaccine) **(A)**; in the Wallonia-Brussels region of Belgium versus FOV with the 2vHPV vaccine **(B)**; and as part of a national HPV vaccine catch-up program compared with FOV with the 9vHPV vaccine in Belgium **(C)** over 100 years ^a^Sensitivity analyses assessed ICER with 9vHPV GNV vs 2vHPV FOV, and 9vHPV GNV vs 9vHPV FOV with: a genital warts incidence rate of 76 per 100,000 (low) and 250 per 100,000 (high); lower (-20%) and higher (+20%) incidence of HPV-related cancers; label-only indications for the 9vHPV vaccine (genital warts, precancerous lesions [CIN 1/2/3, VIN 2/3, VaIN 2/3, anal intraepithelial neoplasia 1/2/3], cervical, vulvar, vaginal, and anal cancers for females; and genital warts and anal cancer for males); duration of protection of 20 years; vaccine price discount of ±10%; and utilities of ±10%.

GNV with the 9v HPV vaccine is a cost-effective strategy versus FOV with the 9vHPV vaccine in the Flanders region, with an ICER of €8,062 per QALY. Sensitivity analyses projected that decreasing the VCR improves ICER, with a 50% VCR leading to an ICER of €4,042 per QALY, whereas boosting the VCR to 95% increases the ICER to €8,854 per QALY (Figure 2A). Decreasing the duration of protection of the 9vHPV vaccine (assumed to be lifelong in the base case) to 20 years would have minimal impact on the ICER (€8,024 per QALY). Assuming a high genital wart incidence will decrease the ICER to €6,974 per QALY, whereas assuming a low incidence will increase the ICER to €8,376 per QALY. Limiting the model to only diseases included in the 9vHPV vaccine label leads to an ICER of €40,847 per QALY (Figure 2A).

### Regional Vaccination Program in Wallonia-Brussels

In the Wallonia-Brussels region, GNV with the 9vHPV was also estimated to provide greater cumulative reductions in HPV6/11/16/18/31/33/45/52/58-related disease compared with FOV with the 2vHPV vaccine (Table 5). The model projected that GNV with the 9vHPV vaccine would decrease the cumulative incidence of anal, penile, and head and neck cancers in males by 294 (17.7%), 206 (20.8%), and 1,391 (18.0%) cases, respectively. In females, an additional 2,025 cases of cervical cancer (16.4%), 7,601 cases of CIN1 (32.3%), 10,522 cases of CIN 2/3 (29.3%), and 12,117 cases of HPV 6/11-related CIN1 (61.6%) would be prevented relative to FOV with the 2vHPV vaccine. Cumulative incidence of genital warts was projected to decrease by 73,421 (63.1%) and 119,708 (65.2%) cases in males and females, respectively.

The model also projected the greatest number of averted deaths would be for head and neck cancer (688 for males and 48 for females), cervical cancer (525), anal cancer (56 for males and 53 for females), and penile cancer (29) (Table 6).

In the Wallonia-Brussels region, GNV with the 9vHPV vaccine was estimated to result in a 13.4% (€32,379,773) reduction in HPV6/11/16/18/31/33/45/52/58-related disease costs after 100 years compared with FOV with the 2vHPV vaccine (Table 7); 23.4 and 65.6% of the reductions in cumulative HPV 6/11/16/18/31/33/45/52/58-related disease costs were attributable to HPV 16/18- and HPV 6/11-related disease costs, respectively.

GNV with the 9v HPV vaccine is a cost-effective strategy versus FOV with the 2vHPV vaccine, with an ICER of €4,179 per QALY, well below previously estimated ICERs for FOV conducted in 2007 (€33,000) (Thiry et al., 2019). Sensitivity analyses projected that decreasing the VCR improves ICER, with a 30% VCR leading to an ICER of €3,180 per QALY, whereas boosting the VCR to 90% increases the ICER to €7,386 per QALY (Figure 2B). Decreasing the duration of protection of the 9vHPV vaccine (assumed to be lifelong in the base case) to 20 years increases the ICER to €6,990 per QALY. Assuming a high genital wart incidence will decrease the ICER to €2,790 per QALY, whereas assuming a low incidence will increase the ICER to €4,446 per QALY. Limiting the model to only diseases included in the 9vHPV vaccine label leads to an ICER of €8,882 per QALY, well below the thresholds commonly used for cost-effectiveness evaluations (Figure 2B) (National Institute for Health and Care Excellence, 2014; Bertram et al., 2016; Thiry et al., 2019).

### National Catch-Up HPV Vaccination Reimbursement Program

Catch-up HPV vaccination with the 9vHPV vaccine through the Belgian national program in both males and females demonstrated greater cumulative reductions in HPV 6/11/16/18/31/33/45/52/58-related diseases relative to FOV with the 9vHPV vaccine over a 100-year period (Table 5), reducing the projected incidence of cervical cancer and penile cancer by 6.8 and 22.1%. Furthermore, additional decreases in HPV-related disease incidence for head and neck cancer and anal cancer were projected in both males (18.8 and 17.8%, respectively) and females (7.1 and 7.4%, respectively). A GNV catch-up strategy with the 9vHPV vaccine was also estimated to reduce genital warts incidence by 44.6 and 30.3% in males and females, respectively, relative to FOV.

The model estimated that the greatest number of averted HPV-related cancer deaths were for head and neck cancer (1,379 [17.8%] for males and 120 [6.4%] for females) and cervical cancer (594 [5.8%]) after 100 years of catch-up GNV relative to FOV with the 9vHPV vaccine (Table 6).

In accordance with the projected reductions in HPV-related disease burdens associated with the catch-up GNV relative to FOV with the 9vHPV vaccine, the model projected a 7.1% reduction (€42,208,242) in HPV 6/11/16/18/31/33/45/52/58-related disease costs over a 100-year period (Table 7). Including only HPV-related diseases that are in HPV vaccine labels in the model resulted in a similar projected 6.9% decrease (€28,632,227) in HPV 6/11/16/18/31/33/45/52/58-related disease costs; 46.4 and 50.6% of the reductions in cumulative HPV 6/11/16/18/31/33/45/52/58-related disease costs were attributable to HPV 16/18- and HPV 6/11-related disease costs.

Catch-up GNV with the 9v HPV vaccine is a cost-effective strategy versus FOV with the 9vHPV vaccine, with an ICER of €6,127 per QALY. Sensitivity analyses projected that decreasing the VCR improves ICER, with a 5% VCR leading to an ICER of €2,866 per QALY, whereas boosting the VCR to 70% increases the ICER to €8,990 per QALY (Figure 2C). Decreasing the duration of protection of the 9vHPV vaccine (assumed to be lifelong in the base case) to 20 years increases the ICER to €8,522 per QALY. Assuming a high genital wart incidence will decrease the ICER to €5,043 per QALY, whereas assuming a low incidence will increase the ICER to €6,361 per QALY. Limiting the model to only diseases included in the 9vHPV vaccine label leads to an ICER of €16,259 per QALY, still below the thresholds commonly used for cost-effectiveness evaluations (Figure 2C) (National Institute for Health and Care Excellence, 2014; Bertram et al., 2016; Thiry et al., 2019).

## Discussion

This analysis included evaluation of the health impact (i.e., HPV-related disease incidence and death) and cost-effectiveness of HPV GNV with 9vHPV vaccine on HPV-related diseases based on recommendations from the Superior Health Council of Belgium as well as other health technology assessment agencies (KCE, 2019; Conseil Supérieur de la Santé, 2017; Health Information and Quality Authority, 2018). Using a validated transmission dynamic model, GNV with the 9vHPV vaccine, whether as part of the regional immunization programs in Flanders and Wallonia-Brussels or the Belgian national catch-up HPV vaccination program, is projected to reduce HPV-related disease incidence and health care costs relative to FOV with either the 9vHPV or the 2vHPV vaccine. Based on the model outcomes, vaccinating boys is projected to reduce anal cancer and genital wart incidence in males, and also provide additional protection for females with substantial reductions in the incidence of cervical cancer, precancerous cervical lesions, anal cancer, and genital warts. Although HPV vaccines are not indicated for the prevention of RRP, head and neck cancer, and penile cancer as per label (Gardasil, 2018), there is evidence demonstrating the efficacy of HPV vaccines in reducing persistent HPV infections at these anatomic sites (e.g., persistent external genital infection (Giuliano et al., 2011), persistent oral infection (Herrero et al., 2013; Wilkin et al., 2018), and many countries and international literature considered that HPV vaccines may protect against these diseases, (Takla et al., 2018; Burger et al., 2014; Chesson et al., 2016; Joint committee on Vaccination and Immunization, 2018; Mauz et al., 2018; Boiron et al., 2016; Matsuzaki et al., 2020). Our model suggests GNV with the 9vHPV vaccine is a cost-effective strategy in Flanders (vs. FOV with the 9vHPV vaccine), Wallonia-Brussels (vs. FOV with the 2vHPV vaccine), and the Belgium national catchup program (vs. FOV with the 9vHPV vaccine), with the ICERs being €8,062, €4,179, and €6,127 in the three programs, respectively. The difference in the ICERs of the Flanders and Wallonia-Brussels regions is the result of the different VCRs assumed in our model based on the VCRs of the current vaccination programs in these regions (approximately 90 and 36% in the Flanders and Wallonia-Brussels regions, respectively) (Tjalma et al., 2018). The sensitivity analyses demonstrated that switching to GNV with the 9vHPV vaccine is cost-effective regardless of the VCR situation analyzed (30–95% for the regional programs and 5–70% for the national catch-up program).

Although there is no official cost-effectiveness threshold in Belgium, (Cleemput et al., 2008a; Cleemput et al., 2012) a threshold value of €33,000 per QALY gained was used in a recent KCE cost-effectiveness analysis of HPV vaccination in Belgium (Thiry et al., 2019). This value falls between the World Health Organization threshold range of 1 to 3 times the per capita gross domestic product (GDP) of the country per additional Disability Adjusted Life Years (Belgium, €37,532 to €112,597) and the United Kingdom National Institute for Health and Care Excellence threshold range of cost-effectiveness (£20,000 to £30,000, or €23,000 to €34,000 per additional QALY) (National Institute for Health and Care Excellence, 2014; Bertram et al., 2016; Thiry et al., 2019). ICERs predicted by our model for Flanders, Wallonia-Brussels, and the Belgian national GNV catchup program were well below these thresholds, thus highlighting that GNV with the 9vHPV vaccine is a very cost-effective strategy in Belgium.

Our model includes all HPV-related diseases (i.e., cervical cancer [including CIN 1/2/3 and carcinoma *in situ*], vaginal cancer [including VaIN 1/2/3], vulvar cancer [including VIN 1/2/3], anal cancer, head and neck cancer, and penile cancer), although sensitivity analyses show that GNV with the 9vHPV is still cost-effective relative to FOV with either the 2vHPV or the 9vHPV vaccines when only considering indications in the label. As per label, HPV vaccines are not indicated for the prevention of RRP, head and neck cancer, and penile cancer (Gardasil, 2018).

The reductions in RRP projected in these analyses are of interest. RRP is a rare, chronic disease associated with severe morbidity and in some cases mortality (Fortes et al., 2017). HPV types 6 and 11 are responsible for more than 90% of cases of RRP (Donne et al., 2010; Fortes et al., 2017). The age distribution for RRP is bimodal, with a juvenile form likely due to exposure to HPV in the peripartum period and an adult form caused by HPV infection in adulthood (Fortes et al., 2017). A systematic review looking at RRP incidence rates in Norway reported an adult rate of 0.23 per 100,000 and 0.88 per 100,000 in females and males, respectively, and a juvenile incidence of 0.09 per 100,000 in females and 0.26 per 100,000 in males (Omland et al., 2012). Furthermore, RRP has no ICD code, which makes it difficult to identify cases and to measure its incidence. Real-world data indicate a benefit of HPV vaccination for preventing and treating RRP (Yiu et al., 2019; Novakovic et al., 2018). In Australia, an HPV vaccination catch-up program with the 4vHPV vaccine has been available since 2007 to all schoolgirls aged 12–13 years, with a catch-up program for older schoolgirls and women up to 26 years of age; schoolboys were subsequently added to the vaccination program in 2013 (Donovan and Callander, 2018). As a result, a clear reduction in the incidence of RRP of at least 8 times between 2012 and 2016 was demonstrated in a prospective study evaluating the impact of this national HPV vaccination program (Novakovic et al., 2018). Furthermore, a retrospective review of adults undergoing treatment for RRP in the United States observed that HPV vaccination was associated with an increase in time between RRP-related surgical procedures and a decrease in the number of procedures required per year (Yiu et al., 2019). Therefore, HPV vaccination has the potential to substantially reduce the burden of RRP.

The impact of HPV vaccination on head and neck cancers is supported by real-world data showing that vaccination is associated with reduced prevalence of oropharyngeal infection with HPV-16, (Mehanna et al., 2018) which accounts for >80% of HPV-related oropharyngeal cancers (de Sanjose et al., 2018).

Regarding penile cancer, there is limited clinical data on the impact of HPV vaccination. In an analysis of five randomized double-blind clinical trials of the 9vHPV vaccine in both females and males, no cases (0/1,394) of penile intraepithelial neoplasia occurred among males in the 9vHPV arm versus four cases (4/1,404) in the control arm (Gardasil, 2018).

The public health impact and cost-effectiveness of GNV with the 9vHPV vaccine has been previously assessed using different transmission dynamic models calibrated to other European settings, including Austria, Italy, Germany, Spain, and France (Mennini et al., 2017; Boiron et al., 2016; Largeron et al., 2017; De La Fuente et al., 2019; Majed et al., 2021). Similar to our findings, these studies reported additional percent reductions in the incidence of cervical cancer over a 100-year period when GNV with the 9vHPV vaccine was compared to FOV with the 4vHPV vaccine (approximately 17–24%) (Mennini et al., 2017; Boiron et al., 2016; Largeron et al., 2017; De La Fuente et al., 2019) or FOV with the 9vHPV vaccine (approximately 3 to 7%) (Mennini et al., 2017; Largeron et al., 2017; De La Fuente et al., 2019; Majed et al., 2021). Additional percent reductions in the incidence of genital warts over a 100-year period ranged from approximately 7 to 29% for females and from approximately 14 to 44% for males were observed when GNV with the 9vHPV vaccine was compared to FOV with either the 4vHPV or 9vHPV vaccine (Mennini et al., 2017; Largeron et al., 2017; De La Fuente et al., 2019; Majed et al., 2021); these reductions were less pronounced than the findings of our analyses, which reported further reductions of approximately 30–65% for females and approximately 45 to 63% for males across the three scenarios assessed. Finally, GNV with the 9vHPV vaccine was found to be a cost-effective strategy relative to FOV with the 4vHPV or 9vHPV vaccine, with predicted ICERs (€13,541–€30,426) falling below or very close to the reference cost-effectiveness thresholds (€30,000 or €40,000) used for these studies (Mennini et al., 2017; Boiron et al., 2016; Largeron et al., 2017; De La Fuente et al., 2019; Majed et al., 2021). Taken together, these studies along with our findings in the Belgian setting demonstrate the potential for a GNV approach with the 9vHPV vaccine to reduce the burden of HPV-associated disease and be a cost-effective strategy in the European setting.

### Limitations

Several limitations are associated with this modeling approach. The model did not assess possible changes to cervical cancer screening methods over the course of the 100 years. The model also did not assess indirect costs, the impact on fertility, as well as neonatal morbidity and mortality due to cervical lesions. In addition, we did not conduct multivariate deterministic sensitivity analyses or probabilistic sensitivity analyses. Currently, disease transmission among populations at high risk, such as MSM and HIV populations, is not included in the model, which may result in underestimating the added benefit of male vaccination compared with an FOV strategy. The presence of groups at high risk can decrease the sensitivity to parameter values, which can lead to relatively small changes in the prevalence of infection despite large changes in transmission rates and infectious periods (Datta et al., 2018). Nevertheless, groups at high risk, such as MSM, often have higher rates of sexually transmitted infections and are thus an important source of infection to the population that must be captured by modeling studies (Datta et al., 2018). In England, selective vaccination of MSM with the 4vHPV vaccine is projected to reduce the incidence of anogenital warts and male HPV-related cancer and is likely to be cost-effective (Lin et al., 2017). Therefore, the inclusion of MSM in future modeling studies will likely increase the benefit of a GNV strategy. Furthermore, the model may overestimate herd immunity, or the protective effects of vaccination at a population level, even among those who were not vaccinated, by assuming coverage in 90% of women for the Flanders scenario. No migration into Belgium was assumed and the model is not able to account for the historical mixed schedule (i.e., 2vHPV and 4vHPV in different regions). In addition, the model incorporated the impact of the vaccine on HPV 31/33/45/52/58 types that cause cervical and anal cancer only and not HPV-related diseases such as vulvar, vaginal, and head and neck cancers. As a result of this exclusion, cases avoided for other cancers and subsequently the value of the 9vHPV vaccine are underestimated. Finally, the amount of underreporting of cancer cases and subsequent deaths due to undiagnosed cancers is not well understood. The model assumes that the death rate for undiagnosed cancer is the same as that of diagnosed cancers. The QALYs gained due to deaths avoided includes deaths due to both diagnosed and undiagnosed cancer and may overestimate the benefit of vaccination.

### Conclusion

Compared with an FOV strategy, a GNV strategy with the 9vHPV vaccine is projected to provide substantial public health (i.e., reductions in HPV-related disease incidence and death) and economic benefits in Belgium when incorporated into the national catch-up program, as well as independent regional vaccination programs in Flanders and Wallonia-Brussels. As a result, a GNV strategy will provide greater benefit in the prevention of HPV-related diseases in the entire male population and additional benefits in females. GNV with the 9vHPV vaccine may also be considered a cost-effective strategy relative to FOV with a broad range of vaccine coverage rates in the regional and national vaccination programs. Future research will look to expand analyses to include populations at high risk, such as MSM, which will truly capture the full impact of a GNV strategy.

## Data Availability

All relevant data is contained within the article. The original contributions presented in the study are included in the article/Supplementary Material; further inquiries can be directed to the corresponding author.
