# Supplementary material for: Health Impact and Cost-Effectiveness of Implementing Gender-Neutral Vaccination With the 9-Valent Human Papillomavirus Vaccine in Belgium
Source: Front Pharmacol. 2021 Apr 12;12:628434. doi: 10.3389/fphar.2021.628434 (PMC8072375; doi:10.3389/fphar.2021.628434)
Supplement: Supplementary file 1 [file table1.docx]

# **SUPPLEMENTARY INDEX**

## **Supplementary Table 1.** Model Input Variables.

| **DEMOGRAPHIC VARIABLES** | | |  |
| --- | --- | --- | --- |
| **Population size, n** | | | **Data Source** |
| **Belgium** | **Female** | 5,754,083 | *Population par lieu de résidence, nationalité, état civil, âge et sexe, 2016. http://bestat.economie.fgov.be/BeStat/.* |
|  | **Male** | 5,568,005 |  |
|  | **Total** | 11,322,088 |  |
| **Wallonia-Brussels** | **Female** | 2,477,116 |  |
|  | **Male** | 2,367,168 |  |
|  | **Total** | 4,844,284 |  |
| **Flanders** | **Female** | 3,276,967 |  |
|  | **Male** | 3,200,837 |  |
|  | **Total** | 6,477,804 |  |

| **Annual all-cause mortality rates for the general population, %** | | | **Data Source** |
| --- | --- | --- | --- |
| **Age group (years)** | **Males** | **Females** | *Tables de mortalité et espérance de vie. 2016, SPF Économie - Direction générale Statistique. https://statbel.fgov.be/fr/themes/population/mortalite-et-esperance-de-vie/tables-de-mortalite-et-esperance-de-vie#figures.* |
| **<1** | 0.00391 | 0.00233 |  |
| **1–8** | 0.00012 | 0.00013 |  |
| **9–10** | 0.00009 | 0.00002 |  |
| **11–12** | 0.00007 | 0.00005 |  |
| **13–14** | 0.00011 | 0.00005 |  |
| **15–17** | 0.00015 | 0.00011 |  |
| **18** | 0.00044 | 0.00023 |  |
| **19** | 0.00062 | 0.00025 |  |
| **20–24** | 0.00058 | 0.00020 |  |
| **25–26** | 0.00064 | 0.00023 |  |
| **27–29** | 0.00069 | 0.00035 |  |
| **30–34** | 0.00086 | 0.00039 |  |
| **35–39** | 0.00107 | 0.00055 |  |
| **40–44** | 0.00167 | 0.00088 |  |
| **45–49** | 0.00238 | 0.00161 |  |
| **50–54** | 0.00392 | 0.00257 |  |
| **55–59** | 0.00658 | 0.00385 |  |
| **60–64** | 0.01100 | 0.00629 |  |
| **65–69** | 0.01687 | 0.00896 |  |
| **70–74** | 0.02510 | 0.01385 |  |
| **75–79** | 0.04202 | 0.02415 |  |
| **80–84** | 0.07171 | 0.04693 |  |
| **>85** | 0.16058 | 0.13424 |  |
| **SEXUAL BEHAVIOR VARIABLES** | | |  |
| **Percent of the population in each of the following sexual activity categories** | | | **Data source** |
| **Sexual activity category** | **Males** | **Females** | *(Data from France): Bajos N, Bozon M. Enquête sur la sexualité en France. Pratiques, genre et santé. Paris, La découverte, 2008, page 240.* |
| **Low** (mean number of sexual partners per year: 0**–**1) | 87.71 | 92.85 |  |
| **Medium** (mean number of sexual partners per year: 2–4) | 9.66 | 6.38 |  |
| **High** (mean number of sexual partners per year: 5+) | 2.63 | 0.77 |  |
| **Mean number of sexual partners per year by activity category and sex** | | | **Data Source** |
| **Sexual activity category** | **Males** | **Females** | *(Data from France): Bajos N, Bozon M. Enquête sur la sexualité en France. Pratiques, genre et santé. Paris, La découverte, 2008, page 240.]* |
| **Low (mean number of sexual partners per year: 0–1)** | 0.86 | 0.84 |  |
| **Medium (mean number of sexual partners per year: 2–4)** | 2.65 | 2.58 |  |
| **High (mean number of sexual partners per year: 5+)** | 8.04 | 7.93 |  |
| **Mean number of sexual partners per year by age group and sex** | | | **Data Source** |
| **Age group (years)** | **Males** | **Females** | *(Data from France): Bajos N, Bozon M. Enquête sur la sexualité en France. Pratiques, genre et santé. Paris, La découverte, 2008, page 240.* |
| **13–14** | 0.0499 | 0.0778 |  |
| **15–17** | 0.62 | 0.97 |  |
| **18–19** | 1.30 | 1.20 |  |
| **20–24** | 1.50 | 1.20 |  |
| **25–29** | 1.40 | 1.20 |  |
| **30–34** | 1.40 | 1.10 |  |
| **35–39** | 1.20 | 1.10 |  |
| **40–44** | 1.10 | 1.00 |  |
| **45–49** | 1.30 | 1.00 |  |
| **50–54** | 1.20 | 0.90 |  |
| **55-59** | 1.20 | 0.80 |  |
| **60–64** | 0.90 | 0.80 |  |
| **65–69** | 0.90 | 0.60 |  |
| **≥70** | 0.60 | 0.40 |  |

| **Sexual mixing among members of different age cohorts** | | | | **Data Source** |
| --- | --- | --- | --- | --- |
| **Ages** | | | **Mixing** | *(US data): Elbasha EH, Dasbach EJ. Impact of vaccinating boys and men against HPV in the United States.* Vaccine*. 2010;28(42):6858-6867.* |
| **Between debut and cessation** | | | 0.40 |  |
| **After cessation** | | | 0.10 |  |
| **SCREENING VARIABLES** | | | | |
| **Cervical cancer screening** | | | | **Data Source** |
| **Percent of females receiving a follow-up screening test after abnormal PAP smear diagnosis** | | | 90.21 | *Calculations based on:*  *Cervical cancer screening program and human papillomavirus (HPV) testing, part II: update on HPV primary screening. KCE Federaal Kenniscentrum voor de Gezondheidszorg - Centre fédéral d'expertise des soins de santé. AND*  *Expert survey in Belgium in 2007* |
| **Percent of females receiving gynecological cancer screening tests at least once every 3 years** | | | 53.70 |  |
| **Percent of females screened for cervical cancer in the past year** | | | | **Data Source** |
| **Age group (years)** | **% of females screened for cervical cancer** | | | *Calculations based on:*  *Cervical cancer screening program and human papillomavirus (HPV) testing, part II: update on HPV primary screening. KCE Federaal Kenniscentrum voor de Gezondheidszorg - Centre fédéral d'expertise des soins de santé.* |
| **0–17** | **0.00** | | |  |
| **18** | **4.38** | | |  |
| **19** | **8.89** | | |  |
| **20–24** | **32.64** | | |  |
| **25–26** | **47.84** | | |  |
| **27–29** | **36.26** | | |  |
| **30–34** | **47.84** | | |  |
| **35–39** | **45.61** | | |  |
| **40–44** | **43.14** | | |  |
| **45–49** | **39.25** | | |  |
| **50–54** | **31.68** | | |  |
| **55–59** | **22.72** | | |  |
| **60–64** | **17.83** | | |  |
| **65–69** | **2.42** | | |  |
| **70–74** | **1.32** | | |  |
| **75–79** | **0.66** | | |  |
| **80–84** | **0.25** | | |  |
| **>85** | **0.09** | | |  |
| **Cervical Screening:  Diagnostic performance of PAP screening and colposcopy** | | | | **Data Source** |
| **PAP specificity** | **0.94** | | | *(US data): Elbasha EH, Dasbach EJ. Impact of vaccinating boys and men against HPV in the United States.* Vaccine*. 2010;28(42):6858-6867.* |
| **Colposcopy sensitivity** | **0.96** | | |  |
| **Colposcopy specificity** | **0.48** | | |  |
| **CIN Screening:  Diagnostic performance of PAP screening** | | | | **Data Source** |
| **CIN stage** |  | | | *(data from France): National Agency for Accreditation and Evaluation of Health (ANAES). « Recommandations pour la pratique clinique : conduite à tenir devant un frottis anormal du col de l’utérus. 1998 et actualisation 2002.* [*http://www.has-sante.fr/portail/upload/docs/application/pdf/frottis_final_fiche_de_synth _350se_2006_11_20__19_46_10_585.pdf*](http://www.has-sante.fr/portail/upload/docs/application/pdf/frottis_final_fiche_de_synth%20_350se_2006_11_20__19_46_10_585.pdf) |
| **CIN 1** | **0.63** | | |  |
| **CIN 2** | **0.61** | | |  |
| **CIN 3** | **0.61** | | |  |
| **DISEASE and TREATMENT VARIABLES** | | | | |
| **Percent of female population with cervical cancer recognizing their symptoms and seeking treatment** | | | | **Data Source** |
| **Cervical cancer stage** | | | |  |
| **Local disease** | **4.1** | | | *Values estimated through model calibration* |
| **Regional disease** | **14.0** | | |  |
| **Distant disease** | **90.0** | | |  |
| **CIN/CIS cases treated** | | | | **Data Source** |
| **Cervical cancer stage** | | | |  |
| **CIN 1** | **15.4** | | | *Values estimated through model calibration* |
| **CIN 2** | **92.19** | | |  |
| **CIN 3** | **96.74** | | |  |
| **CIS** | **96.74** | | |  |
| **Female population with vaginal cancer recognizing their symptoms and seeking treatment** | | | | **Data Source** |
| **Local disease** | **3.8** | | | *Values estimated through model calibration* |
| **Regional disease** | **18.0** | | |  |
| **Distant disease** | **90.0** | | |  |
| **VaIN/CIS cases treated** | | | | **Data Source** |
| **VaIN 1** | **30.0** | | | *Values estimated through model calibration* |
| **VaIN 2/3** | **100.0** | | |  |
| **CIS** | **100.0** | | |  |
| **Female population with vulvar cancer recognizing their symptoms and seeking treatment** | | | | **Data Source** |
| **Vulvar cancer stage** | | | | *Values estimated through model calibration* |
| **Local disease** | **3.8** | | |  |
| **Regional disease** | **18.0** | | |  |
| **Distant disease** | **90.0** | | |  |
| **VIN/CIS cases treated** | | | | **Data Source** |
| **VIN 1** | **N/A** | | | *Values estimated through model calibration* |
| **VIN 2/3** | **N/A** | | |  |
| **CIS** | **N/A** | | |  |
| **Annual recurrent respiratory papillomatosis mortality rate** | | | | **Data Source** |
| **Male** | 0.01 | | | **Values used in US model** |
| **Female** | 0.01 | | |  |
| **Percent of genital warts treated** | | | | **Data Source** |
| **Male** | **66.4** | | | *Values estimated through model calibration* |
| **Female** | **51.4** | | |  |
| **Population with anal cancer recognizing their symptoms and seeking treatment** | | | | **Data Source** |
| **Anal cancer stage** | **Males** | **Females** | | *Values estimated through model calibration* |
| **Local disease** | 9.0 | **4.0** | |  |
| **Regional disease** | 30.0 | **9.0** | |  |
| **Distant disease** | 90.0 | **90.0** | |  |
| **Male population with penile cancer recognizing their symptoms and seeking treatment** | | | | **Data Source** |
| **Penile cancer stage** | | | | *Values estimated through model calibration* |
| **Local disease** | **13.0** | | |  |
| **Regional disease** | **28.0** | | |  |
| **Distant disease** | **90.0** | | |  |
| **Population with head and neck cancer recognizing their symptoms and seeking treatment** | | | | **Data Source** |
| **Head and neck cancer stage** | **Males** | **Females** | | *Values estimated through model calibration* |
| **Local disease** | 1.0 | **5.0** | |  |
| **Regional disease** | 12.0 | **16.0** | |  |
| **Distant disease** | 90.0 | **90.0** | |  |
